# Comparison of islet isolation result and clinical applicability according to GMP‐grade collagenase enzyme blend in adult porcine islet isolation and culture

**DOI:** 10.1111/xen.12703

**Published:** 2021-06-26

**Authors:** Kyungmin Kwak, Jae‐kyung Park, Joohyun Shim, Nayoung Ko, Hyoung‐Joo Kim, Yongjin Lee, Jun‐Hyeong Kim, Michael Alexander, Jonathan R. T. Lakey, Hyunil Kim, Kimyung Choi

**Affiliations:** ^1^ Optipharm Inc. Cheongju Korea; ^2^ Department of Biomedical Engineering University of California Irvine Irvine CA USA; ^3^ Department of Surgery University of California Irvine Orange CA USA

**Keywords:** adult porcine islets, collagenase, digestion enzymes, islet isolation, islet yield, type 1 diabetes

## Abstract

**Background:**

Porcine islet xenotransplantation is a promising treatment for type 1 diabetes as an alternative to human pancreatic islet transplantation and long‐term insulin therapy. Several research groups have explored porcine islets as an alternative to the inconsistent and chronic shortage of pancreases from human organ donors. Studies have confirmed successful transplant of porcine islets into non‐human primate models of diabetes; however, in most cases, they require more than one adult porcine donor to achieve sufficient viable islet mass for sustained function. The importance of GMP‐grade reagents includes the following: specific enzymes utilized in the pancreatic isolation process were identified as a key factor in successful human clinical islet transplantation trials using cadaveric islets. As xenotransplantation clinical research progresses, isolation reagents and digestion enzymes play a key role in the consistency of the product and ultimately the outcome of the islet xenotransplant. In this study, we evaluated several commercially available enzyme blends that have been used for islet isolation. We evaluated their impact on islet isolation yield and subsequent islet function as part of our plan to bring xenotransplantation into clinical xenotransplantation trials.

**Methods:**

Adult porcine islets were isolated from 16 to 17‐month‐old Yucatan miniature pigs following standard rapid procurement. Pigs weighed on average 48.71 ± 2.85 kg, and the produced pancreases were 39.51 ± 1.80 grams (mean ± SEM). After ductal cannulation, we evaluated both GMP‐grade enzymes (Collagenase AF‐1 GMP grade and Liberase MTF C/T GMP grade) and compared with standard non‐GMP enzyme blend (Collagenase P). Islet quality control assessments including islet yield, islet size (IEQ), membrane integrity (acridine orange/propidium iodide), and functional viability (GSIS) were evaluated in triplicate on day 1 post‐islet isolation culture.

**Results:**

Islet yield was highest in the group of adult pigs where Collagenase AF‐1 GMP grade was utilized. The mean islet yield was 16 586 ± 1391 IEQ/g vs 8302 ± 986 IEQ/g from pancreases isolated using unpurified crude Collagenase P. The mean islet size was higher in Collagenase AF‐1 GMP grade with neutral protease than in Collagenase P and Liberase MTF C/T GMP grade. We observed no significant difference between the experimental groups, but in vitro islet function after overnight tissue culture was significantly higher in Collagenase AF‐1 GMP grade with neutral protease and Liberase MTF C/T GMP grade than the crude control enzyme group. As expected, the GMP‐grade enzyme has significantly lower endotoxin levels than the crude control enzyme group when measured.

**Conclusions:**

This study validates the importance of using specifically blended GMP grade for adult pig islet isolation for xenotransplantation trials and the ability to isolate a sufficient number of viable islets from one adult pig to provide a sufficient number for islets for a clinical islet transplantation. GMP‐grade enzymes are highly efficient in increasing islet yield, size, viability, and function at a lower and acceptable endotoxin level. Ongoing research transplants these islets into animal models of diabetes to validate in vivo function. Also, these defined and reproducible techniques using GMP‐grade enzymes allow for continuance of our plan to advance to xenotransplantation of isolated pig islets for the treatment of type 1 diabetes.

## 1 INTRODUCTION

Diabetes mellitus (diabetes) is a chronic disease affecting over 463 million people globally, as recently reported by the International Diabetes Federation (IDF).^1^ Type 1 diabetes (T1DM) is an autoimmune disease in which beta cells are completely destroyed, resulting in metabolic dysfunction as a consequence of insufficient circulating levels of insulin.^2^, ^3^ Insulin therapy (exogenous insulin) is a principle method of treatment, but it does not mimic exactly the physiology of insulin secretion in the body.^4^ The alternative is pancreas transplantation and/or pancreatic islet cell transplantation. However, the severe shortage of available cadaveric donors limited the widespread adoption of this transplantation.^5^ In addition, the number of pancreases and islet quality decreased by several factors, including increasing donor age and obesity.^6^ For these reasons, many researchers studied alternative source of islets to solve these key supply problems.

Xenotransplantation using pig islets is one of the candidates for clinical transplantation into diabetic patients. Successful xenotransplantation from pigs to humans provides a potential unlimited supply of tissues and cells, resolving the shortage of donor tissues for transplantation.^7^ Several research groups studied islet xenotransplantation using pigs as the source of insulin‐producing cells.^8^ There has been considerable research exploring the use of purified enzyme blends in islet isolation that have been specifically developed for islet isolation in an attempt to improve consistency and increase yields and function of isolated islets.^9^ Since 2006, a total of 9 groups performed non‐human primate experiments using porcine islets. In 2015, Shin JS et al^10^ reported on islets isolated from two pigs transplanted into 4‐ to 6‐kg non‐human primates (NHPs). Porcine islets were isolated using Liberase MTF C/T GMP‐grade enzymes. They also used Cobra Venom Factor (CVF), anti‐TNF‐alpha monoclonal antibody and ATG induction, and sirolimus immunosuppression, in addition to anti‐CD154. 3 of 5 recipients also received ex vivo expanded regulatory T‐cell therapy. Islet grafts functioned for 180, 180, 503, 513, and over 603 days as defined by the presence of porcine c‐peptides.^10^ Recently, Bottino et al transplanted porcine islets into non‐human primates.^11^ They isolated islets using CIzyme™ Collagenase MA and BP protease (Viacyte). The islet yield was 1000 IEQ/g‐2237 IEQ/g, and islet function was using the GSIS method, the stimulation index ranged from 2.0 to 10.8.^11^ Bottino et al used mycophenolate mofetil and anti‐CD154–based immunosuppression with multi‐transgenic islet donors in their study. Islet graft function was 0, 3, 5, 160, and 365 days for the 5 recipients in their study. As repeatedly shown, islet quality is very important for success of transplantation.^11^, ^12^, ^13^ Both studies used adult pig islets and demonstrated good quality but required two or more pigs to achieve the transplantable dose for their small transplant recipients. This issue will become even more apparent in clinical trials in larger sized human patients. Nonetheless, these studies are in line with the concept that highest quality islets are an essential starting criterion, as shown for human islet transplantation,^13^ and further illustrate the additional complexity and limitation of islet xenotransplantation. Following these studies, the selection of enzyme in the isolation process is critical because it affects to islet quality such as the survival rate, viability, and function and reproducibility of isolations.

Manufacturing highly functioning, viable porcine islets involves establishing consistent isolation and digestion techniques and also needs a suitable stable enzyme type and blends of enzymes.^14^ In 2015, good manufacturing practice (GMP)–grade enzyme (Liberase MTF C/T GMP grade, Roche) was shown to increase islet yield in human islet isolation while addressing the key issue of GMP manufacturing and storage of the enzyme products. GMP‐grade enzyme is needed to adhere to GMP guidelines for clinical trials.^15^ Before Liberase MTF C/T GMP‐grade, crude enzymes were used for human and animal pancreatic islet isolations, but these enzymes could not be utilized for clinical trials because of lot‐to‐lot variation, enzyme impurity, and high endotoxin.^16^, ^17^ When comparing human islet isolation using crude enzymes and GMP‐grade enzymes, GMP‐grade enzymes resulted in higher yield than crude enzyme formulations.^16^ These islets were able to restore blood glucose control within 7 days when transplanted into diabetic nude mice.^18^ Recently, several GMP‐grade enzymes are being developed. O’ Gorman D et al^19^ reported that they confirmed similar islet yield, viability, and function in human islet isolation using Collagenase NB‐1 GMP‐grade enzymes, developed by Serva, and Liberase MTF C/T GMP‐grade, manufactured by Roche.

Following these studies, we selected collagenase with neutral protease (called Collagenase AF‐1 GMP grade) and collagenase with thermolysin (called Liberase MTF C/T GMP grade). As control enzymes, Roche Collagenase P was used because of its historical use in porcine and rodent islet isolations.^20^, ^21^, ^22^, ^23^

In this study, we aim to measure the outcome of utilizing GMP‐grade enzymes and confirm whether isolated islets are suitable for clinical transplantation. We hypothesize that GMP‐grade enzymes will have significant consistency and improve islet isolation yield, viability, size distribution, and function compared with crude enzymes.

## 2 MATERIALS AND METHODS

### 2.1 Islet isolation: procurement and digestion

Porcine pancreases were obtained from adult 12‐ to 18‐month‐old Yucatan miniature pigs (OptiPharm Inc)(18.12 ± 1.77 months) of either sex. All animal procedures were performed under a protocol approved by OptiPharm Institutional Animal Care and Use Committee (OPTI‐IAC‐19003). The pancreas was rapidly excised after dissection around the pancreas while maintaining perfusion (mean within 20 minutes). The pancreases weighed 39.51 ± 1.80 g (mean ± SEM) and were preserved in cold HTK solution (Essential Pharma) on ice until digestion using a Ricordi digestion chamber. The average cold ischemia time from removal to initiation of enzyme perfusion was 55 (55.23 ± 5.92) min. The pancreases were distended via the pancreatic duct using the aforementioned enzymes, removed fat and vessels, and were divided into 8‐10 pieces. The enzyme used for digestion was Collagenase P (Roche, Indianapolis, IN, USA), Collagenase AF‐1 GMP grade with neutral protease (Nordmark Biochemicals, Uestersen, Germany) and Liberase MTF C/T GMP grade (Roche). Information about the donor and islet isolation procedure is presented in Table 1. All enzymes were dissolved in 1X HBSS for a minimum of 30 minutes (Corning 21‐023‐CV) and filtered through a 0.22‐um filter at room temperature and stored in 4°C. Islet isolation was conducted in accordance with the modified pancreas dissociation method.^24^, ^25^ We determined the digestion time using DTZ staining. During digestion, we sampled digested tissues, stained by DTZ, and monitored using a microscope. If an intact islet was detected, we stopped digestion and changed cold washing media. After digestion, digests were centrifuged at 200 *g* for 5 minutes and collected in a 1‐L sterile bottle on ice with 1X HBSS added 10% FBS (Hyclone). After collection of pancreatic digest and counting islets in duplicate from a representative sample of the tissue, the digested tissue was incubated for 1 hour at 4°C in HTK solution before purification with gentle mixing of the tissue every 10‐15 minutes.

**TABLE 1:** Information about the donor (porcine) and islet isolation procedure

Species	Yucatan miniature pig		
Enzyme type	Collagenase P (n = 14)	Collagenase AF‐1 GMP grade (n = 8)	Liberase MTF C/T GMP grade (n = 6)
Age (Mo)	17.83 ± 2.2	18.38 ± 3.95	18.33 ± 4.2
Body weight (kg)	47.69 ± 4.79	48.98 ± 5.56	50.73 ± 2.67
Pancreas weight (g)	36.74 ± 2.43	41.06 ± 4.08	43.9 ± 2.62
Digestion time (min)	9.35 ± 0.57	9.62 ± 0.70	12 ± 0.81^a^
Enzyme			
Collagenase (Unit)	332.9 ± 24.72	593.9 ± 59.12	697.0 ± 65.02
T or NP (Unit)	Not used	NP: 31.68 ± 3.151	T: 93 884 ± 12 439

Note

All islet isolations were from pancreases isolated from Yucatan miniature pigs at OPTIPHARM CO LTD, Korea. The age of the donor pigs was 17‐18 mo, and body weight average was 48 kg. Following standard enzyme loading, porcine pancreas were enzymatically and mechanically digested with similar, non‐significantly digestion times

Abbreviations: NP, neutral protease; T, thermolysin.

*P* value < .1.

^a^ Compared significant with Collagenase P and each enzyme labeled.

### 2.2 Islet purification

Islets were purified with a continuous gradient using a COBE 2991 cell processor (Terumo BCT). Continuous density gradient composed of HBSS solution and high‐density Ficoll (Biocoll, Biochrome AG Seromed). The continuous gradient was centrifuged at 2000 rpm for 7 minutes. Gradient samples (1‐10) were stained with dithizone (DTZ, Sigma‐Aldrich) to confirm the fraction with an intact islet. Fractions with intact islets were centrifuged at 90 *g* for 1 minute with no brake and washed.^26^

### 2.3 Islet culture

After purification, islets were cultured in CMRL 1066–supplemented media (Corning Cellgro) with 10% heat‐inactivated fetal bovine serum (Hyclone) at 37°C in a 5% CO_2_ incubator.

### 2.4 Islet yield, islet size and viability

Aliquots were made before and after purification and 24‐hour tissue culture. The islets were counted using an islet number and islet equivalent number (IEQ) after staining with DTZ (Sigma‐Aldrich).^27^ Islet sizes were determined by calculating the average size of islets. Islet viability after 24‐hour culture was measured using double‐florescence staining with acridine orange (Sigma)/propidium iodide (Sigma) for 30 minutes: islet viability: 100% ‐ (dead islet cells/total islet cells × 100%).^28^

### 2.5 Endotoxin test using isolated islet

Endotoxin concentrations were determined using a Pierce LAL Chromogenic Endotoxin Quantitation kit (Thermo Fisher Scientific). Isolated islets were sonicated for 30 seconds in ice. Lysed cells were incubated for 10 minutes on ice and tested for endotoxin level following the manufacturer's protocol.

### 2.6 Islet function: glucose stimulated insulin secretion

A duplicate sample of hand‐counted 10 islets were placed in a 24‐well plate (Corning Cellgro) with a Millicell filter insert (Merck Millipore) and incubated at 37°C and 5% CO_2_ for 1 hour in each media: low glucose rinse (60 mg/dL), low glucose (60 mg/dL), and then high glucose (300 mg/dL) in Krebs‐Ringer bicarbonate buffer (KRBB).^29^ The supernatant was collected in labeled tubes and stored at −20°C until analysis. Insulin levels were measured using a porcine insulin enzyme‐linked immunosorbent assay (Porcine Insulin ELISA; cat# MBS738643, Mybiosource Inc.) and measured with a microplate reader (Tecan and Magellan V7). The stimulation index (SI) is the insulin concentration released from high glucose divided by the insulin concentration released from low glucose.^30^

### 2.7 Statistical analysis

All data were presented as mean ± standard error of mean (SEM). The statistical significance of the differences between two pairs of groups was analyzed by one‐way ANOVA. A level of *P* < .05 was accepted as significant. Statistical analyses were conducted using GraphPad Prism 5 software (GraphPad Software, Inc.).

## 3 RESULTS

### 3.1 Islet yield comparison of the enzymes

**FIGURE 1:**
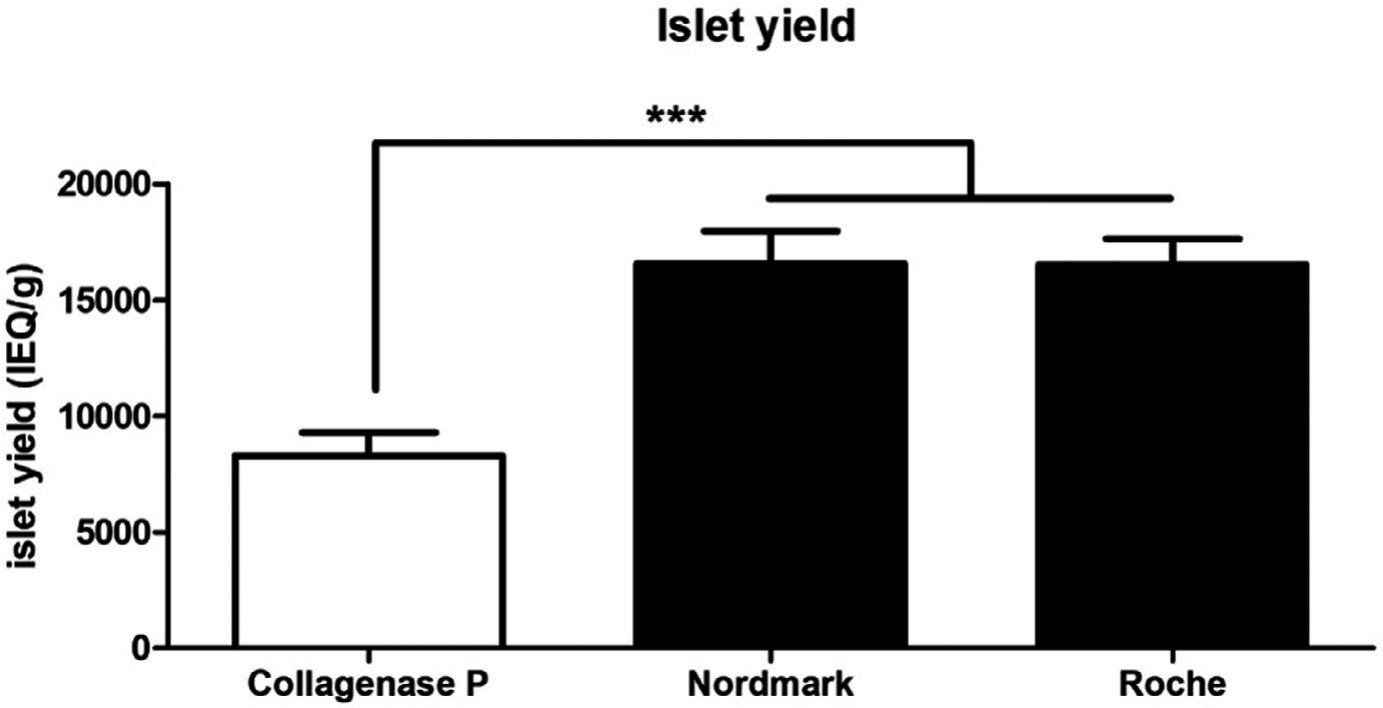
islet yield comparison. Porcine islets were isolated using each enzyme and islet yield measured per gram of pancreas tissue. Islet yield was significantly increased compared to collagenase P, but Nordmark and Roche (Liberase MTF) has no significant difference in islet yield. (Collagenase P: 8302 ± 986, Nordmark: 16 586 ± 1391, Roche: 16 517 ± 1127) (*: *P* < .1, **: *P* < .01, ***: *P* < .001)

To compare islet yield between enzyme groups, an islet equivalent (IE) was counted on day 1 of tissue culture at 37°C. After day 1 culture, porcine islets isolated with Collagenase P produced on average 8302 ± 986 IEQ/g (n = 14), with a total of 337 400 ± 42 341 IEQs (n = 14) (mean ± SEM). Pig islets isolated with Collagenase AF‐1 GMP grade with neutral protease produced a mean yield of 16 586 ± 1391 IEQ/g (n = 8), with a total of 723 013 ± 48 871 IEQs (n = 8) (mean ± SEM) (*P* < .05). Collagenase AF‐1 GMP grade with neutral protease isolations produced a significantly higher yield of islets than Collagenase P (*P* < .001). In the Liberase MTF C/T GMP grade group, a mean yield was 16 517 ± 1127 IEQ/g (n = 6) and total IEQ was 683 369 ± 82 839 (Figure 1). Compared with Collagenase P and Collagenase AF‐1 GMP grade, Liberase MTF C/T GMP grade islet isolations had significantly higher yield than Collagenase P, but there was no significant difference compared with Collagenase AF‐1 GMP grade with neutral protease.

### 3.2 Islet size comparison of the enzymes

**FIGURE 2:**
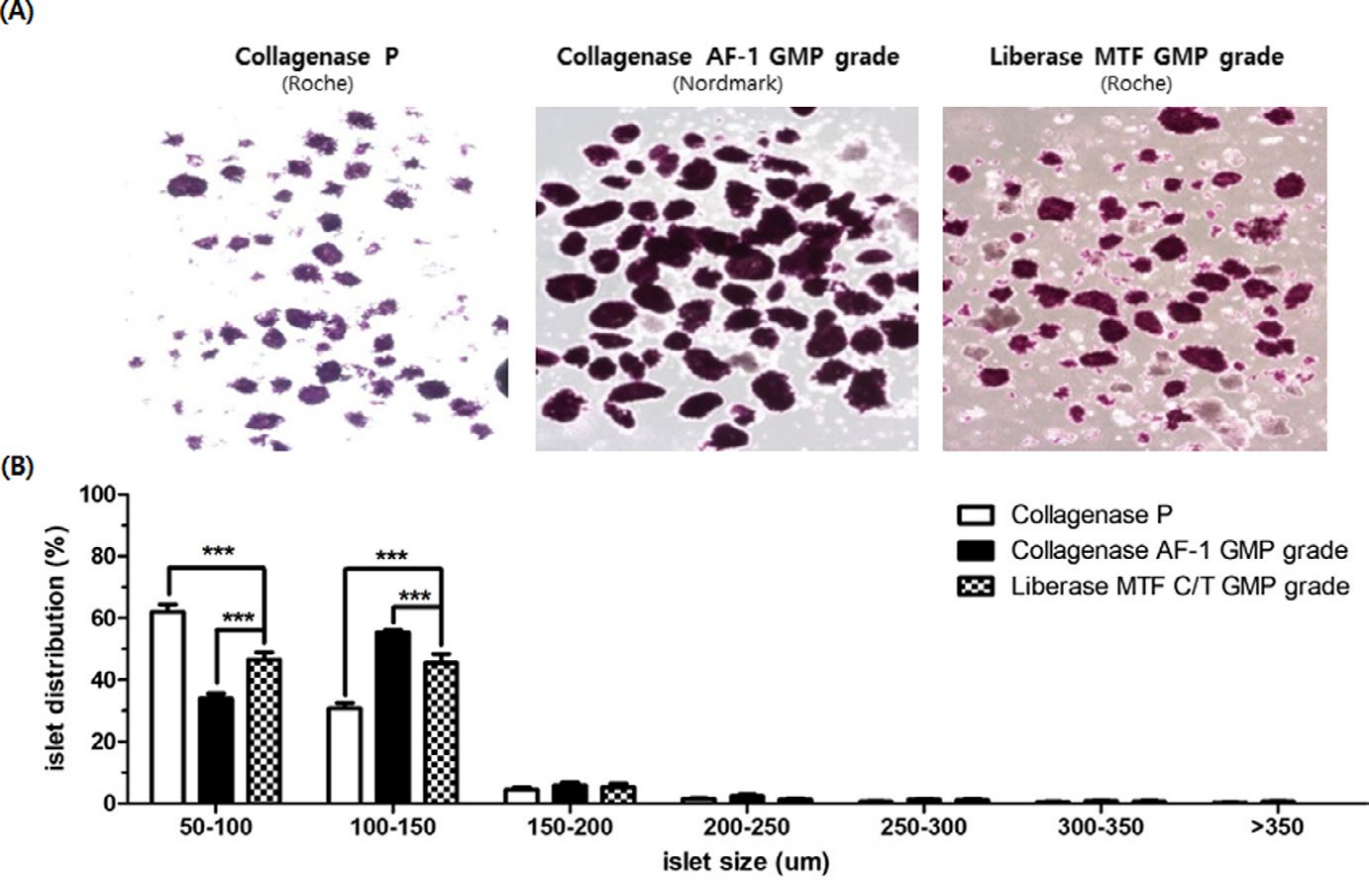
Islet morphology monitoring (X40) and islet cell size analyze through DTZ staining. A, After 1 d of tissue culture, we monitored islet morphology after DTZ staining. Our result showed that GMP grade enzyme has a larger proportion of larger islets compare with islets isolated from the crude enzyme. In islets isolated using GMP grade enzyme, Nordmark enzyme resulted in a higher proportion of larger islets than Liberase MTF C/T GMP grade. B, Islet size distribution categories were analyzed and showed that islets isolated Collagenase AF‐1 GMP grade enzyme (Nordmark) had a lower distribution in 50‐100 um cell sized islets compared with Collagenase P and Liberase MTF C/T GMP grade isolated islets. Smaller islets are indicative of damage and fragmentation of the islets. (*: *P* < .1, **: *P* < .01, ***: *P* < .001)

By comparing islet size distribution between the enzymes, Collagenase P had significantly increased amount of smaller islets (50 ~ 100 μm) compared with isolation performed using Collagenase AF‐1 GMP grade with neutral protease and Liberase MTF C/T GMP grade (Collagenase P: 62 ± 2.5%, Collagenase AF‐1 GMP grade with neutral protease: 38.3 ± 5.9%, Liberase MTF C/T GMP grade: 45.0 ± 3.38%, *P* < .001). Islets of size 100 ~ 150 μm produced using Collagenase AF‐1 GMP grade with neutral protease had significantly a higher ratio than Collagenase P and collagenase with thermolysin (Collagenase AF‐1 GMP grade: 51.7 ± 5.2%, Collagenase P: 30.5 ± 1.8%, Liberase MTF C/T GMP grade: 44.7 ± 2.0%, *P* < .001) (Figure 2). There was no significant difference in islet numbers >150 μm between these groups in our study.

### 3.3 Viability of Isolated islets

Islet viability was measured in duplicate using acridine orange (AO) and propidium iodide (PI) staining on day 1 of culture following established protocols.^27^ Islet viability was significantly different between Collagenase P (82.7 ± 1.3%), Collagenase AF‐1 GMP grade (89.3 ± 1.21%), but we observed no significant difference in Collagenase AF‐1 GMP grade with Liberase MTF C/T GMP grade islet isolations (85.3 ± 3.42%) (Table 2)(*P* > .05).

**TABLE 2:** Summary of islet yield using GMP‐grade enzymes and crude enzyme

Species	Yucatan miniature pig		
Enzyme type	Collagenase P	Collagenase AF‐1 GMP grade	Liberase MTF C/T GMP grade
Manufacture	ROCHE	NORDMARK	ROCHE
Grade	Crude	GMP	GMP
Islet yield	8302 ± 986 IEQ/g	16 586 ± 1391 IEQ/g^a^	16 517 ± 1127 IEQ/g^a^
Stimulation index	2.07 ± 0.02	4.73 ± 0.23^a^	3.87 ± 0.12^a^ ^,^ ^b^
Viability (%)	82.7 ± 1.3	89.3 ± 1.21^a^	85.3 ± 3.42

Note

Our result showed that the GMP‐grade enzyme has higher yield than crude enzyme. The islet isolation protocol using Liberase MTF was not established, but Collagenase AF‐1 GMP grade with neutral protease has higher yield and stimulation index than Liberase MTF.

*P* value < .1.

^a^ Compared significant with Collagenase P and each enzyme labeled.

^b^ Collagenase AF‐1 GMP grade and each enzyme labeled.

### 3.4 Function of isolated islets

**FIGURE 3:**
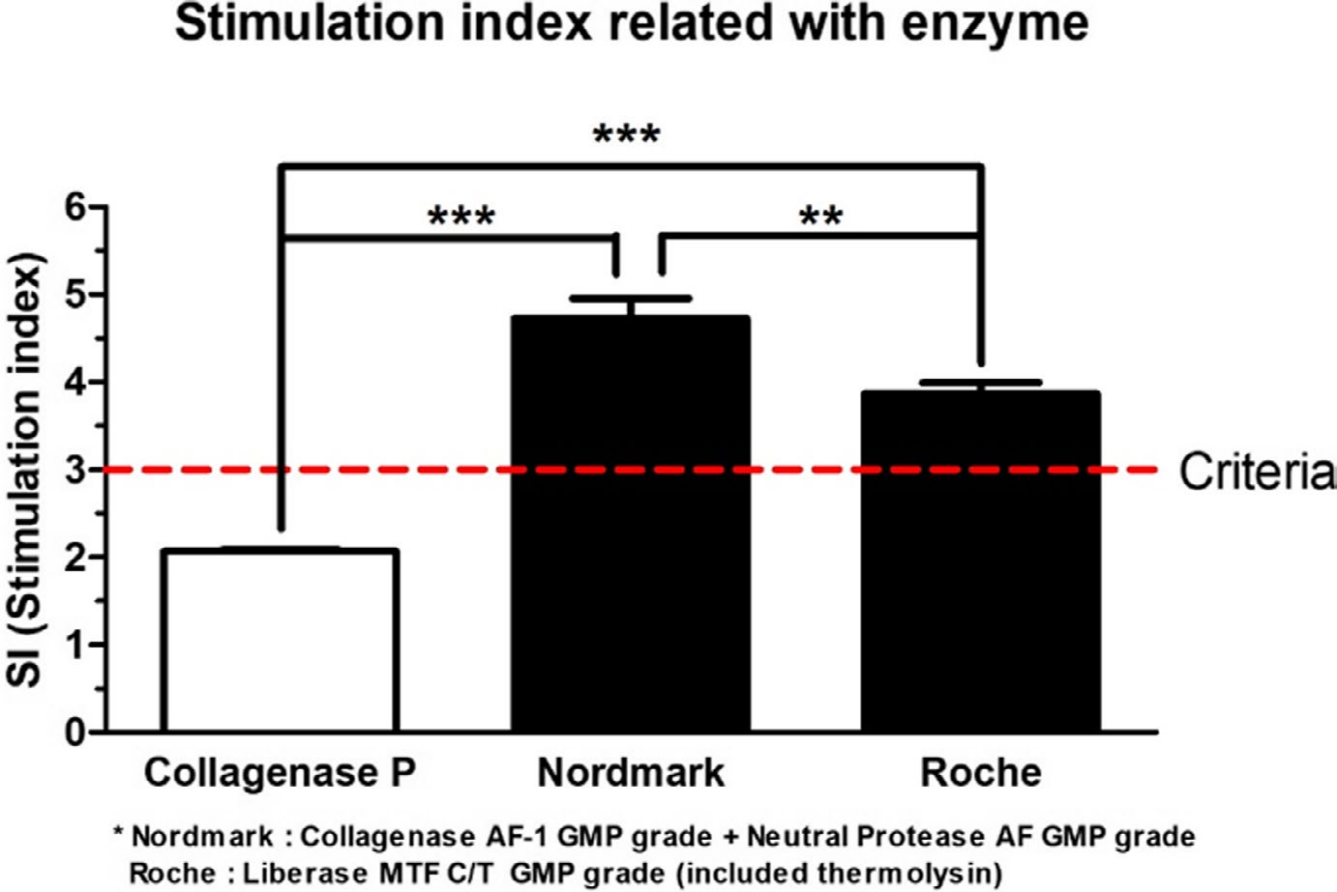
Glucose stimulation index using isolated islet. After 1 d culture, glucose stimulated insulin secretion (GSIS) was performed on the islets, stimulation index presented as ratio of insulin secreted in high glucose over that in low glucose. Both Nordmark and Roche enzymes resulted significantly higher stimulation index compared to Collagenase P, meeting the SI > 3 criterion. (*: *P* < .1, **: *P* < .01, ***: *P* < .001)

The glucose‐stimulated insulin secretion (GSIS) method was used to confirm the dynamic functional response of the isolated islets in vitro when islets were stimulated with low concentration (60 mg/dL) and high concentration of glucose (300 mg/dL). The porcine insulin level was measured and analyzed by ELISA and represented as stimulation index (SI) values. After day 1 of culture at 37°C, the islets isolated using Collagenase AF‐1 GMP grade with neutral protease had a calculated SI of (4.73 ± 0.23) and Liberase MTF C/T GMP grade isolated islet (3.87 ± 0.12) were significantly higher than those isolated using Collagenase P enzyme (2.07 ± 0.02, *P* < .001). When comparing islets isolated using Collagenase AF‐1 GMP grade with neutral protease and Liberase MTF C/T GMP grade, Collagenase AF‐1 GMP grade with neutral protease yielded significantly higher than Liberase MTF C/T GMP grade enzyme (*P* < .01) (Figure 3).

### 3.5 Endotoxin level

**FIGURE 4:**
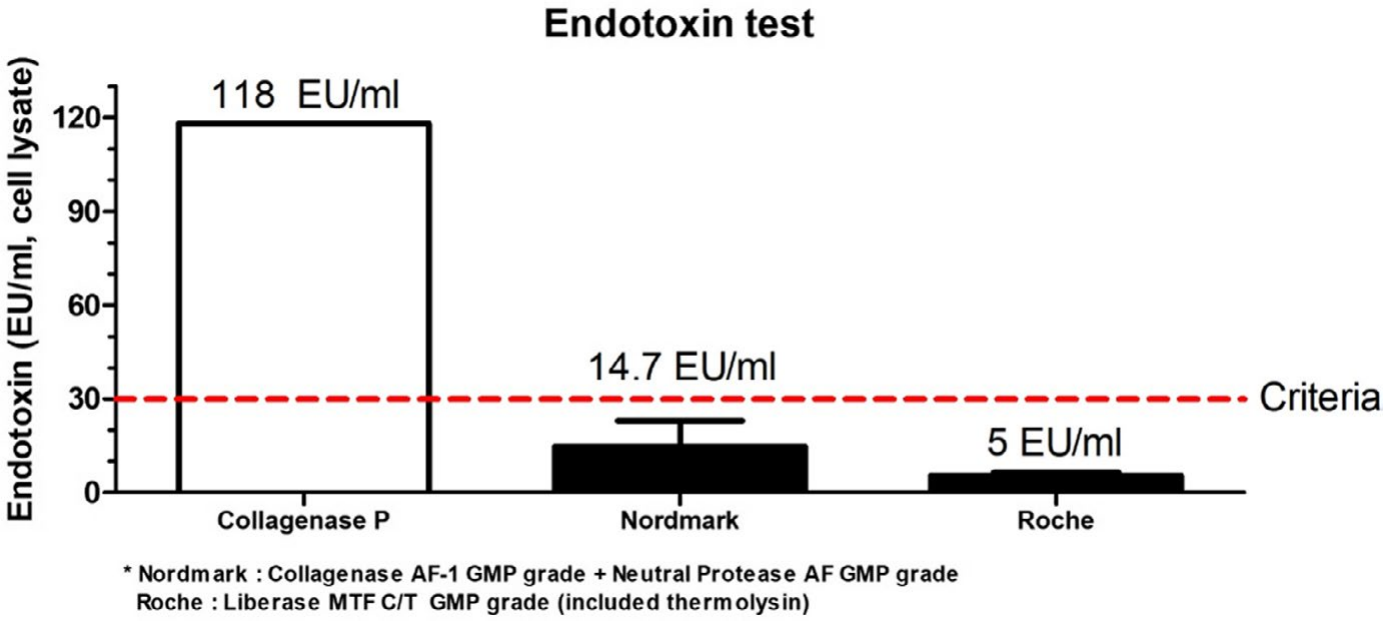
Endotoxin level of isolated islets. Endotoxin level was measured in islet cell lysate after isolation. Both Nordmark and Roche enzyme resulted in significantly lower endotoxin level compared to Collagenase P, meeting the criteria of <30 EU/mL

Endotoxin level analysis was performed using an Endotoxin ELISA kit (Thermo Pierce LAL endotoxin ELISA kit) using the manufacturers’ protocols. For measuring endotoxin contents, buffer ≤ 0.001 EU/mL (Endosafe LAL reagent water) was used. In Collagenase P, the endotoxin level was 118 EU/mL. The endotoxin level using Collagenase AF‐1 GMP grade with neutral protease AF GMP grade was 14.7 ± 8.159 EU/mL (n = 4) (mean ± SEM), whereas that using Liberase MTF C/T GMP grade was 5.4 ± 1.04 EU/mL (n = 3) (mean ± SEM) (Figure 4).

## 4 DISCUSSION

Xenotransplantation has been developed to supplement the limitation of human allo‐islet transplantation for treatment type 1 diabetes mellitus (T1D). Several parameters in the islet isolation process can affect the production of successful islet yield, viability, and function of the isolated islets. The result of islet quality decided the success of islet transplantation results.^31^ Some scientific articles reported that islet yield, viability, cost, and function varied with pig age.^5^, ^32^, ^33^ Lakey et al^5^ compared cost and quality of isolated islets of juvenile, neonatal, and adult pigs. In their study, they found that the neonatal porcine islet cell cluster (NPCC) has high insulin secretory function but lower islet yield. Based on this study, our group performed islet isolation based on varying pig age. We found that the yield of isolated islets was higher in 12‐ to 18‐month‐old pig (data not shown). For porcine islet transplantation into humans, the minimum dose is over 10 000 IEQ/kg per type 1 diabetic patient.^34^ This effective dose may even be higher when using pig islets. If the patient needed more islets, islet isolation from 2 or 3 porcine may be necessary^5^ because of the lower insulin secretion for cure diabetes in porcine islets. This has been previously reported by Mueller et al^35^ who found that when they performed glucose‐stimulated insulin secretion profiles using human and adult porcine islets, human islets had an higher insulin level under high glucose stimulation. For these results, enzyme quality plays a crucial role in producing a large number of islets.^17^ Before this enzyme comparison study, we isolated porcine islets using collagenase type IV with only limited yield (5002 ± 282.1 IEQ/g; data not shown). To confirm the composition of GMP‐grade enzymes suitable for porcine islet isolation, islet isolation was performed by changing the ratio of collagenase and thermolysin (or neutral protease) (data not shown). As a result, we used the optimal collagenase and thermolysin (or neutral protease) ratio, as shown in Table 1. Following this pilot study, we isolated porcine islets using crude enzymes (Collagenase P) and GMP‐grade enzymes (Collagenase AF‐1 GMP grade with neutral protease and Liberase MTF C/T GMP grade). Adult pig islet yield was higher when using GMP‐grade enzymes than crude enzymes. This result showed that GMP‐grade enzymes can provide sufficient islet yield for future clinical trials of islet xenotransplantation.

In 2007, Lehmann et al reported that the islet size is a key factor in determining the human islet transplantation outcome.^36^, ^37^ They used a perfusion assay using small islets (defined as a diameter between 50 and 150 μm) and large islets (defined as a diameter between 150 and 300 μm) and found that small islets have a higher insulin level than large islets. In this study, Collagenase AF‐1 GMP grade with neutral protease has a higher ratio of 100‐ to 150‐μm size islets than Collagenase P and Liberase MTF C/T GMP grade (Figure 2). We measured the insulin secretion level using the GSIS method and calculated the stimulation index (Figure 3). Some researchers reported that porcine islets have a low insulin level compare with human islets, but one researcher reported that human and adult porcine islets have similar total insulin levels.^35^, ^38^ In our study, the calculated stimulation index in isolated pig islets using GMP‐grade enzymes (SI > 3.0) was significantly higher than those in crude collagenase enzymes (SI < 3.0, *P* value < .001). When we analyzed data from Graham et al's and ours, we confirmed that isolated porcine islets are capable of secreting a similar insulin level as human islets. Also, islets isolated using Collagenase AF‐1 GMP grade with neutral protease had significantly higher stimulation index than those isolated using Liberase MTF C/T GMP grade. We believe these data support that GMP‐grade enzymes are the optimal enzymes for islet isolation in clinical trials. GMP‐grade enzymes allow for consistency and confidence in the porcine islet production and islet recovery when used in clinical trials.

For clinical islet transplantation, the endotoxin level also plays an important role in the death of islets after transplantation.^39^ In 2017, Wang et al^34^ reported that the endotoxin content needed was less than 5 EU/kg recipient body weight. And in 2001, Berney et al^40^ reported that the endotoxin level of the enzyme related to the islet survival–induced apoptosis and necrosis. In that study, they used collagenase type V and Liberase HI for mouse islet isolation and transplanted the islets into diabetic mice. The islet transplant group that received a high level of endotoxin islets delayed the diabetes reversal time.^40^ Following that study, we analyzed the endotoxin level in isolated islets and islet‐cultured media. Endotoxin was not measured in islet‐cultured media (data not shown). Islets isolated using GMP‐grade enzymes have a lower endotoxin level than those isolated using crude enzymes (Figure 4). Our result showed that our isolated islets meet the optimal criterion for use in clinical trials for islet xenotransplantation. Further investigation on islet transplantation into diabetic mice or NHPs using the aforementioned enzymes needs to be accomplished for clinical trials.

Islet quality is very important for transplantation into diabetic patients. It has been reported that the quality of islet affects various characteristics such as enzyme quality including grade and islet isolation technology.^41^, ^42^, ^43^, ^44^ It is also important to find the ratio of collagenase and thermolysin (or neutral protease) for islet isolation.^45^, ^46^, ^47^ In our group, we used GMP‐grade enzymes to increase islet yield and confirmed viability over 80%. When the function was confirmed with the glucose‐stimulated insulin secretion (GSIS) method, stimulation index (SI) value was confirmed over 3.0.

In conclusion, our group confirmed that using rapid pancreas procurement, immediate cell processing, and our islet isolation protocol, the adult porcine pancreas provides enough isolated viable islets that could be used for islet xenotransplantation in a 60‐kg diabetic patient and could have an equivalent level of function as reported for human islets. We also found that GMP‐grade enzymes have higher islet isolation yield than crude enzymes. We believe that this method is a reliable and reproducible technique for porcine islet isolation using GMP‐grade enzymes, and it will be of great help in islet xenotransplantation for the treatment of type 1 diabetes.

## References

[1] Patterson CC, Karuranga S, Salpea P, et al. Worldwide estimates of incidence, prevalence and mortality of type 1 diabetes in children and adolescents: results from the international diabetes federation diabetes atlas, 9th edition. Diabetes Res Clin Pract. 2019;157:107842. 31518658 10.1016/j.diabres.2019.107842

[2] Roep BO, Thomaidou S, van Tienhoven R, Zaldumbide A. Type 1 diabetes mellitus as a disease of the β‐cell (do not blame the immune system?). Nat Rev Endocrinol. 2021;17(3):150‐161. 33293704 10.1038/s41574-020-00443-4 PMC7722981

[3] Kawasaki E. Type 1 diabetes and autoimmunity. Clin Pediatr Endocrinol. 2014;23:99‐105. 25374439 10.1297/cpe.23.99 PMC4219937

[4] Bellin MD, Dunn TB. Transplant strategies for type 1 diabetes: whole pancreas, islet and porcine beta cell therapies. Diabetologia. 2020;63:2049‐2056. 32894315 10.1007/s00125-020-05184-7

[5] Vanderschelden R, Sathialingam M, Alexander M, Lakey JRT. Cost and scalability analysis of porcine islet isolation for islet transplantation: comparison of juvenile, neonatal and adult pigs. Cell Transplant. 2019;28:967‐972. 31037984 10.1177/0963689719847460 PMC6719497

[6] Schuetz C, Anazawa T, Cross SE, et al. β cell replacement therapy: the next 10 years. Transplantation. 2018;102(2):215‐229. 28885496 10.1097/TP.0000000000001937

[7] Ching CD, Harland RC, Collins BH, Kendall W, Hobbs H, Opara EC. A reliable method for isolation of viable porcine islet cells. Arch Surg. 2001;136:276‐279. 11231845 10.1001/archsurg.136.3.276

[8] Coe TM, Markmann JF, Rickert CG. Current status of porcine islet xenotransplantation. Curr Opin Organ Transplant. 2020;25:449‐456. 32773503 10.1097/MOT.0000000000000794 PMC8279426

[9] Corrales N, Park S, Lau H, et al. Comparison of islet characterization from use of standard crude collagenase to GMP‐grade collagenase enzyme blends in preweaned porcine islet isolations. Cell Transplant. 2020;29:1‐11. 10.1177/0963689720977835 PMC7873766 33267618

[10] Shin JS, Kim JM, Kim JS, et al. Long‐term control of diabetes in immunosuppressed nonhuman primates (NHP) by the transplantation of adult porcine islets. Am J Transplant. 2015;15:2837‐2850. 26096041 10.1111/ajt.13345

[11] Bottino R, Wijkstrom M, van der Windt DJ, et al. Pig‐to‐monkey islet xenotransplantation using multi‐transgenic pigs. Am J Transplant. 2014;14:2275‐2287. 25220221 10.1111/ajt.12868 PMC4169326

[12] Rayat GR, Gazda LS, Hawthorne WJ, et al. First update of the international xenotransplantation association consensus statement on conditions for undertaking clinical trials of porcine islet products in type 1 diabetes ‐ chapter 3: porcine islet product manufacturing and release testing criteria. Xenotransplantation. 2016;23(1):38‐45. 26923763 10.1111/xen.12225

[13] Papas KK, Suszynski TM, Colton CK. Islet assessment for transplantation. Curr Opin Organ Transplant. 2009;14:674‐682. 19812494 10.1097/MOT.0b013e328332a489 PMC2859186

[14] Iglesias I, Valiente L, Shiang KD, Ichii H, Kandeel F, Al‐Abdullah IH. The effects of digestion enzymes on islet viability and cellular composition. Cell Transplant. 2012;21:649‐655. 22236690 10.3727/096368911X623826

[15] Qi M, Valiente L, McFadden B, et al. The choice of enzyme for human pancreas digestion is a critical factor for increasing the success of islet isolation. Transplant Direct. 2015;1:1‐9. 10.1097/TXD.0000000000000522 PMC4486320 26146662

[16] Wang Y, Paushter D, Wang S, et al. Highly purified versus filtered crude collagenase: comparable human islet isolation outcomes. Cell Transplant. 2011;20:1817‐1825. 21396158 10.3727/096368911X564994 PMC3759232

[17] Wolters GHJ, Vos ‐Scheperkeuter GH, van Deijnen JHM, van Schilfgaarde R. An analysis of the role of collagenase and protease in the enzymatic dissociation of the rat pancreas for islet isolation. Diabetologia. 1992;35:735‐742. 1324862 10.1007/BF00429093

[18] Linetsky E, Bottino R, Lehmann R, Alejandro R, Inverardi L, Ricordi C. Improved human islet isolation using a new enzyme blend, liberase. Diabetes. 1997;46:1120‐1123. 9200645 10.2337/diab.46.7.1120

[19] O'Gorman D, Kin T, Imes S, Pawlick R, Senior P, Shapiro AMJ. Comparison of human islet isolation outcomes using a new mammalian tissue‐free enzyme versus collagenase NB‐1. Transplantation. 2010;90:255‐259. 20463640 10.1097/TP.0b013e3181e117ce

[20] Kim JW, Sun C, Jeon SY, et al. Glucocorticoid treatment independently affects expansion and transdifferentiation of porcine neonatal pancreas cell clusters. BMB Rep. 2012;45:51‐56. 22281014 10.5483/bmbrep.2012.45.1.51

[21] Eom YS, Gwon AR, Kwak KM, et al. Notch1 has an important role in β‐cell mass determination and development of diabetes. Diabetes Metab J. 2021;45(1):86‐96. 32174059 10.4093/dmj.2019.0160 PMC7850870

[22] Kim JW, Park SY, You YH, et al. Targeting PGC‐1α to overcome the harmful effects of glucocorticoids in porcine neonatal pancreas cell clusters. Transplantation. 2014;97:273‐279. 24448589 10.1097/01.TP.0000438627.68225.25

[23] Georges P, Muirhead RP, Williams L, et al. Comparison of size, viability, and function of fetal pig islet‐like cell clusters after digestion using collagenase or liberase. Cell Transplant. 2002;11:539‐545. 12428743

[24] Ricordi C, Lacy PE, Finke EH, Olack BJ, Scharp DW. Automated method for isolation of human pancreatic islets. Diabetes. 1988;37(4):413‐420. 3288530 10.2337/diab.37.4.413

[25] Ricordi C, Lacy PE, Scharp DW. Automated islet isolation from human pancreas. Diabetes. 1989;38:140‐142. 2642838 10.2337/diab.38.1.s140

[26] Friberg AS, Ståhle M, Brandhorst H, Korsgren O, Brandhorst D. Human islet separation utilizing a closed automated purification system. Cell Transplant. 2008;17:1305‐1313. 19364068 10.3727/096368908787648100

[27] Khiatah B, Qi M, Wu Y, et al. Pancreatic human islets and insulin‐producing cells derived from embryonic stem cells are rapidly identified by a newly developed Dithizone. Sci Rep. 2019;9:5‐10. 31243300 10.1038/s41598-019-45678-y PMC6594947

[28] Salgado M, Gonzalez N, Medrano L, et al. Semi‐automated assessment of human islet viability predicts transplantation outcomes in a diabetic mouse model. Cell Transplant. 2020;29:1‐10. 10.1177/0963689720919444 PMC7586280 32410459

[29] Ning Y, Zhen W, Fu Z, et al. Ranolazine increases β‐cell survival and improves glucose homeostasis in low‐dose streptozotocin‐induced diabetes in mice. J Pharmacol Exp Ther. 2011;337:50‐58. 21228065 10.1124/jpet.110.176396

[30] Zhao W, Zhao SP. Different effects of statins on induction of diabetes mellitus: an experimental study. Drug Des Devel Ther. 2015;9:6211‐6223. 10.2147/DDDT.S87979 PMC4664500 26648697

[31] Balamurugan AN, Naziruddin B, Lockridge A, et al. Islet product characteristics and factors related to successful human islet transplantation from the collaborative islet transplant registry (CITR) 1999–2010. Am J Transplant. 2014;14:2595‐2606. 25278159 10.1111/ajt.12872 PMC4282081

[32] Krishnan R, Truong N, Gerges M, et al. Impact of donor age and weaning status on pancreatic exocrine and endocrine tissue maturation in pigs. Xenotransplantation. 2015;22:356‐367. 26381493 10.1111/xen.12184

[33] Kim JH, Kim H, Lee KW, et al. Influence of strain and age differences on the yields of porcine islet isolation: extremely high islet yields from SPF CMS miniature pigs. Xenotransplantation. 2007;14:60‐66. 17214705 10.1111/j.1399-3089.2006.00364.x

[34] Wang W, Liang Q, Nie W, Zhang J, Chen CH. Xenotransplantation for islets from clinical side. Intech. 2016;13:67.

[35] Mueller KR, Balamurugan AN, Cline GW, et al. Differences in glucose‐stimulated insulin secretion in vitro of islets from human, nonhuman primate, and porcine origin. Xenotransplantation. 2013;20:75‐81. 23384163 10.1111/xen.12022 PMC4145818

[36] Lehmann R, Zuellig RA, Kugelmeier P, et al. Superiority of small islets in human islet transplantation. Diabetes. 2007;56:594‐603. 17327426 10.2337/db06-0779

[37] Ricordi C, Gray DWR, Hering BJ, et al. Islet isolation assessment in man and large animals. Acta Diabetol Lat. 1990;27:185‐195. 2075782 10.1007/BF02581331

[38] Graham ML, Bellin MD, Papas KK, Hering BJ, Schuurman HJ. Species incompatibilities in the pig‐to‐macaque islet xenotransplant model affect transplant outcome: a comparison with allotransplantation. Xenotransplantation. 2011;18:328‐342. 22168140 10.1111/j.1399-3089.2011.00676.x

[39] Górski Ł, Wszoła M, Berman A, Kwiatkowski A, Kuthan R, Chmura A. Clinical estimate of endotoxin levels in human islet cell suspensions destined for transplantation. MEDtube Sci. 2014;2:35‐39.

[40] Berney T, Molano RD, Cattan P, et al. Endotoxin‐mediated delayed islet graft function is associated with increased intra‐islet cytokine production and islet cell apoptosis 1. Transplantation. 2001;71(1):125‐131. 11211177 10.1097/00007890-200101150-00020

[41] Yamamoto T, Horiguchi A, Ito M, et al. Quality control for clinical islet transplantation: organ procurement and preservation, the islet processing facility, isolation, and potency tests. J Hepatobiliary Pancreat Surg. 2009;16:131‐136. 19242650 10.1007/s00534-009-0064-z

[42] Hart NJ, Powers AC. Use of human islets to understand islet biology and diabetes: progress, challenges and suggestions. Diabetologia. 2019;62:212‐222. 30547228 10.1007/s00125-018-4772-2 PMC6325002

[43] Natasha HJN, Wei XT, Ye XK, Adrian T. Human islet isolation and distribution efforts for clinical and basic research. OBM Transplant. 2019;2:31.

[44] Yesil P, Michel M, Chwalek K, et al. A new collagenase blend increases the number of islets isolated from mouse pancreas. Islets. 2009;1:185‐190. 21099271 10.4161/isl.1.3.9556

[45] Kin T, Zhai X, Murdoch TB, Salam A, Shapiro AMJ, Lakey JRT. Enhancing the success of human islet isolation through optimization and characterization of pancreas dissociation enzyme. Am J Transplant. 2007;7:1233‐1241. 17359501 10.1111/j.1600-6143.2007.01760.x

[46] Brandhorst D, Brandhorst H, Johnson PRV. Enzyme development for human islet isolation: five decades of progress or stagnation? Rev Diabet Stud. 2017;14:22‐38. 28632819 10.1900/RDS.2017.14.22 PMC6115004

[47] Brandhorst H, Johnson PR, Mönch J, Kurfürst M, Korsgren O, Brandhorst D. Comparison of clostripain and neutral protease as supplementary enzymes for human islet isolation. Cell Transplant. 2019;28:176‐184. 30419762 10.1177/0963689718811614 PMC6362525

